# Rapid Prototyping Technologies and their Applications in Prosthodontics, a Review of Literature

**Published:** 2015-03

**Authors:** Kianoosh Torabi, Ehsan Farjood, Shahram Hamedani

**Affiliations:** a Dept. of Prosthodontics, School of Dentistry, Shiraz University of Medical Sciences, Shiraz, Iran.; b DDS, MScD. Private Practice, Shiraz, Iran

**Keywords:** CAD-CAM, Three-Dimensional/methods, Computer-aided rapid prototyping model, Computer-aided design, Dentistry

## Abstract

The early computer-aided design/computer-aided manufacturing (CAD/CAM) systems were relied exclusively on subtractive methods. In recent years, additive methods by employing rapid prototyping (RP) have progressed rapidly in various fields of dentistry as they have the potential to overcome known drawbacks of subtractive techniques such as fit problems. RP techniques have been exploited to build complex 3D models in medicine since the 1990s. RP has recently proposed successful applications in various dental fields, such as fabrication of implant surgical guides, frameworks for fixed and removable partial dentures, wax patterns for the dental prosthesis, zirconia prosthesis and molds for metal castings, and maxillofacial prosthesis and finally, complete dentures. This paper aimed to offer a comprehensive literature review of various RP methods, particularly in dentistry, that are expected to bring many improvements to the field. A search was made through MEDLINE database and Google scholar search engine. The keywords; ‘rapid prototyping’ and ‘dentistry’ were searched in title/abstract of publications; limited to 2003 to 2013, concerning past decade. The inclusion criterion was the technical researches that predominately included laboratory procedures. The exclusion criterion was meticulous clinical and excessive technical procedures. A total of 106 articles were retrieved, recited by authors and only 50 met the specified inclusion criteria for this review. Selected articles had used rapid prototyping techniques in various fields in dentistry through different techniques. This review depicted the different laboratory procedures employed in this method and confirmed that RP technique have been substantially feasible in dentistry. With advancement in various RP systems, it is possible to benefit from this technique in different dental practices, particularly in implementing dental prostheses for different applications.

## Search Strategy

This paper aimed to offer a comprehensive literature review of various rapid prototyping (RP) methods, particularly in dentistry, that are expected to bring many improvements to this field. The study was purposed to focus on the technical feasibility of the technique. A search was made through MEDLINE database and Google scholar search engine. The keywords; ‘rapid prototyping’ and ‘dentistry’ were searched in title/abstract of publications; limited to 2003 to 2013, concerning past decade. The inclusion criterion was the technical researches that predominately included laboratory procedures. The exclusion criterion was detailed clinical and excessive technical procedures. References of selected articles were also reviewed for possible inclusion in the study. Titles and abstracts of all selected articles were reviewed and upon identification for possible inclusion, full text of the article was reviewed thoroughly and cross-matched with the predefined inclusion criteria. A total of 106 articles were retrieved, recited by authors and only 50 met the specified inclusion criteria for this review. Selected articles had used rapid prototyping techniques in various fields in dentistry through different techniques. 

## Literature review

The first attempts to automate the production of dental restorations began more than 20 years ago. [1-2] Subsequently, computer-aided design/computer-aided manufacturing (CAD/CAM) technologies were introduced to the dental community in early 1980s. [1] All CAD/CAM systems have three functional components: 1) A digitalization tool/scanner that transforms geometry into digital data that can be processed by a computer. 2) Software which processes scanner data and produces a data set readable by a fabrication machine. 3) A manufacturing technology that takes the data set and transforms it into the desired product by fabricating the restoration. [1-2] To fabricate a physical prototype in industry and/or medicine, two different approaches have been utilized: subtractive and additive. [3] In a subtractive method, material is subtracted from an initial block of material to leave the desired shaped part (such as a dental restoration). [4]

Early CAD/CAM systems relied almost exclusively on cutting a restoration from a prefabricated block with the use of burs, diamonds or diamond disks. [5] This is usually accomplished by conventional numeric control (NC) machining such as milling. [6] Subtractive processes use carefully-planned tool movements to cut material. The NC machining is used typically in small model-making machines for which they are used to fabricate metallic and/or ceramic crowns in dentistry. [7] The subtractive fabrication can create a complete shape effectively, though at the expense of material being wasted. In a typical subtractive method in dentistry, approximately 90 percent of the initial bock is removed to create a typical dental restoration. [1] Torabi *et al.* in their studies concluded that the CAD/CAM system could compete well with conventional systems for clinical fit and fracture resistance and can achieve acceptable results in vitro. [8-9] Vojdani *et al.* [10] compared the marginal and internal fit of metal copings cast from wax patterns fabricated with a CAD/CAM system and the conventional method. Their findings showed that only conventional method could result in copings with clinically acceptable margins of less than 120µm. [10]

Alternatively, additive fabrication is a process in which the final desired part is manufactured by adding multiple layers of material on top of one another. [11] The key idea of this innovative method is that the three dimensional CAD (3D-CAD) model is sliced into many thin layers and the manufacturing equipment uses this geometric data to build each layer sequentially until the part is completed. Hence, additive fabrication is often referred as “layered manufacturing”, “direct digital manufacturing”, “three-dimensional printing”, or “solid freeform fabrication”. [12-13] Additive technology scan yield arbitrarily complex shapes with cavities and undercuts; frequently the case in human anatomy structures. [9-11]

Subtractive methods have some limitations in comparison with additive techniques: The precision fit of the inside contour of the restoration depends on the size of the smallest usable tool for each material and if the cutting tool was larger in diameter than some parts of the tooth preparation, it will result in reduction of internal fit precision or inferior marginal properties.

1. A considerable amount of raw material is wasted because the unused portions of the mono-blocks must be discarded after milling and recycling of the excess ceramic material is not feasible.
2. Milling tools are exposed to heavy abrasion and wear, therefore, withstanding only short running cycles.
3. Microscopic cracks can be introduced into ceramic surfaces due to machining of this brittle material.
4. It is neither easy nor economic for big, full undercuts and/or complex milling parts. [9, 14-16]

Rapid prototyping (RP) techniques, the so-called “generative manufacturing techniques”, exhibit the potential to overcome the described shortages. [17-20] RP simply consists of two phases: virtual phase (modeling and simulating) and physical phase (fabrication). Virtual prototyping is development of model by dynamic and interactive simulation. The course of forming the physical model is formation of 3D physical model by CAD. Some characteristics of the process can be stated as:

Objects can be produced with different geometrical intricacy without involving the setup of the machine or final assembly. Objects can be produced by employing different types of materials such as composites. Moreover, with a controlled conduction, different materials can be used at different places in an object. The construction of complex objects can be fast, convenient and uncomplicated by additive fabrication systems. [12]

The RP techniques have been employed to build complex 3D models in medicine since the 1990s. [21-24] The chief benefit of RP techniques is the medical models that can be produced with undercuts, voids, intricate internal geometrical details and anatomical landmarks such as facial sinuses and neurovascular canals. [15, 25] The RP model is currently employed to improve medical diagnosis and to provide a precise surgical treatment plan. The technique would help shorten the surgery time and consequently reducing the patients’ risk. [15, 26-27]

In current years, RP is becoming more appealing for dental purposes. The innovations in molding materials and forming procedure have improved the RP techniques so that this technology is no longer adopted only for prototyping; it is used for reproduction of real functional elements. [15, 28-29] The feasibility of this technique is increasing in different dental practice fields such as oromaxillofacial surgery and prosthesis, [30-39]production of surgical guide or physical models in dental implant therapies, [40-43] and prosthodontics. [15, 44-45]

The RP techniques can also be employed to plan, produce, and develop dental prostheses such as crowns, fixed and removable partial dentures (FPDs and RPDs) and also copings. This technique would eliminate any faults caused by human skills and intervention in traditional fabrication of dental prosthesis and comparably is time saving. [15, 46-47] Digital dental surveying and RP-produced sacrificial patterns could be accomplished to fabricate RPDs frameworks. [48] Moreover, RP has been used to reduce the extra-oral time which need to be spent in autogenous tooth transplantation. [49] In 2011, Morea *et al.* used the SLA technique for accurate insertion of the orthodontic mini screws. [50]

Yu *et al.* used this technique for producing the pre-surgical nasoalveolar molds in treatment of infants suffering from unilateral cleft lip and palate. [34] The dental practice has also benefited from RP in accurate reconstruction of maxillofacial defects [51-55] and also in osteogenic distraction with satisfactory outcomes. [56-58]

**Classification of RP technologies in dentistry**

The frequent technologies that are adopted in dental practice are stereolithography (SLA), inkjet-based system (3DP), selective laser sintering (SLS), and fused deposition modeling (FDM). While various materials can be employed in these technologies; wax, plastics, ceramics, and metals are commonly used by several studies in dentistry. [7, 12]

**Stereolithography (SLA)**

**Figure 1:**
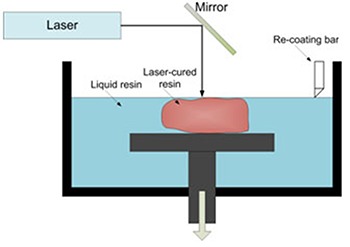
Schematic depiction of SLA

This method includes a photosensitive liquid resin bath, a model-building platform, and an ultraviolet (UV) laser for curing the resin. [7] The layers are cured and bond successively to form a solid object for impression rationales, exploited in reconstructive surgeries and sub-periosteal surgery in dental implant therapies (Figure 1). Fabrication of surgical drilling templates during insertion of dental implants is the current foremost purpose for using SLA models in dental practice. [7] SLA-made surgical drill guides have been proved to benefit from high precision by several well-documented researches. [59-65]

**Inkjet-based system or 3DP**

**Figure 2:**
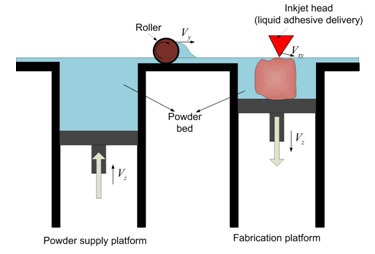
A schematic representation of 3DP technique

In this technique, a measured amount of the raw powder-form material is initially dispensed from a container by a moving piston (Figure 2). A roller then distributes and compresses the powder at the top of the fabrication chamber. A liquid adhesive is then deposited from the multi-channel jetting head in a 2D pattern onto the powder, make it bond and form a layer of the object. When a layer is completed, the piston helps spread and join the next powder layer. This incremental (layer-by- layer) method is gradually continued to achieve a complete built up of prototype. [7] Unbound powder is swept up subsequent to a heating process, leaving the fabricated part sound and intact.

**Selective Laser Sintering (SLS)**

**Figure 3:**
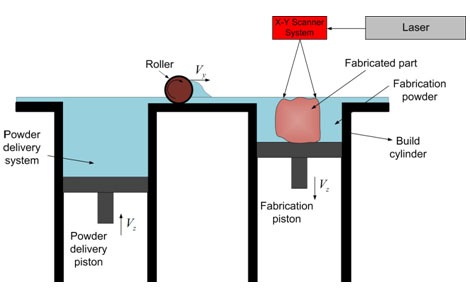
A Schematic image of SLS technique

Figure 3 shows a schematic depiction of the selective laser sintering method (SLS). In SLS method, layers of particular powder material are fused into a 3D model by adopting a computer-directed laser. A roller distributes the powdered material over the surface of a build cylinder. Powder is spread layer-by-layer on top of the preceding hardened layer and sintered repeatedly. [7, 12-13] To hold the new fresh layer of powder, the supporting platform relegates one object layer thickness. The surface of this firmly compressed powder is then exposed to a beam of laser. The procedure is self-sustaining and all parts can be bond layer-by-layer. [7] SLS technique has significant advantages in dentistry, particularly prosthodontics, since various thermoplastic materials such as nylon composite, investment casting wax, metallic materials, ceramics and thermoplastic composites can be used in this method. [7, 45]

**Fused Deposition Modeling (FDM)**

The FDM is a rapid prototyping technique in which a thermoplastic material is extruded layer by layer from a nozzle, controlled by temperature. In this technique, a filament of a thermoplastic polymer material suckles into the temperature-controlled FDM extrusion nozzle dome. It is then heated to a free-flowing semi-liquid form. The motion of the nozzle head is controlled by a processor and traces and deposits the material in extremely thin layers onto a subsidiary platform. The head leads the material into place with an ample precision. A portion of the subject is built up layer by layer and the material solidifies within 0.1s after being ejected from the nozzle and bonds to the layer below. The supporting structures are contrived for overhanging geometries and are later removed by cutting them out from the object. [7]

**The applications of RP techniques in facial and dental prosthesis**

RP techniques are now regarded as a promising alternative for dental prosthesis production. [15] This review particularly focuses on fabrication of wax pattern of prosthesis, all-ceramic crowns, metal prostheses (in clouding FPDs and framework for removal partial dentures) and casts for prostheses.

**Dental prosthesis wax pattern fabrication**

**Figure 4:**
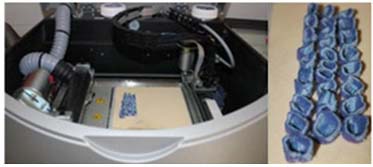
Wax-patterns fabricated by RP technology

With the introduction and attractiveness of RP technology, a new style is possible for automatic wax-up construction [66-67] illustrated in Figure 4. After the wax pattern is fabricated by RP, the traditional lost-wax process is still needed. The process is more affordable than laser melting or sintering direct manufacturing processes, which still remains financially unattainable for most dental laboratories. [15]

**Rapid prototyping of dental (facial) prosthesis mold (shell)**

**Mold (shell) for metal casting**

3D printing produces ceramic casting molds for metal casting using a incremental printing method. [68] With RP techniques many labor-intensive and time-consuming steps of the traditional investment casting technique is eliminated. [15] The technique also skips the process of design and manufacturing of wax and core tooling, wax and core molding, wax assembly, shell dipping and drying, and wax elimination. [15]

**Mold for facial prosthesis**

RP techniques have been employed effectively for fabrication of facial prosthesis over the past decade. [15, 39, 69] Pattern fabrication with, the aid of RP, has been a feasible procedure, although, the conventional flasking and investing procedures were still crucial to make the actual prosthesis. Using a mold would remove the conventional flasking and investment procedures, and would shorten the process of making the prosthesis. [15, 70] Moreover, the generated resin mold can be kept since the mold is long-lasting and allows the pouring in multiple times. [71]

**Mold for complete dentures**

The limited available research articles reveals that advanced manufacturing technologies have not been successfully implemented in this field yet. [72] The technology briefly is comprised of the instituting a 3D graphic record of artificial teeth for parameterization positioning, yielding 3D data of edentulous models and rims in centric relation, finding a CAD route and emergence of a software for complete dentures, fabricating physical flasks (molds) by 3DP, and finishing the complete denture using a traditional laboratory procedure. [15, 73]

**Direct dental metal prosthesis fabrication**

RP technology, particularly selective laser melting (SLM) and selective laser sintering (SLS) technology have been on the focus of attention of scientists for the brisk fabrication of high-precision metal parts with various resources and shapes. [53] Dental prostheses are very appropriate to be processed by employing SLS/SLM technique, regarding their complex geometry and their capability to be customized without the extensive manual pre- or post-processing steps. [15, 74]

**All-ceramic restoration fabrication**

A direct inkjet fabrication process has been anticipated for the fabrication of the green-zirconia all-ceramic dental restoration using a slurry micro extrusion process. [17] This innovative method is a favorable CAD/RP system with great ability to produce all-ceramic dental restorations with high precision, cost competence, and minimum material intake. This method is still in the experimental phase. [17]

In general, the advantages of CAD/CAM technique can be concluded as [1] elimination of disruptions of the impression material since the impression phase is eliminated [2] By using the surface scanner, the model can be produced without the potential possibility of tissue deformation [3] the model is formed from natural tissues, therefore, more accurate-looking prosthesis is obtained [4] less space for storage is need since the models are stored in hard disks. When compared to all these advantages, the most highlighted disadvantage of CAD/CAM technique is its high cost. Even though most of the procedure is accomplished by the computer, proficiency of the clinician in application and coloring would impact the success of the final prosthesis. [75]

## Conclusion

The literature review depicted that rapid prototyping (RP) techniques have been substantially employed in dentistry. A combination of dental sciences and manufacturing technologies is the notion behind use of RP in fabrication of dental prosthesis. Multiple steps should be taken in fabrication of prosthesis or restoration in conventional methods which would abide manual errors and spends lot of time of dentist, laboratory technician and patient to obtain a good fitting prosthesis. With the aid of computer in RP, the numbers of steps are reduced, time is saved and dental models are reconstructed with high level of accuracy, precise form and shape with pertinent reproducibility. [76]

With advancement in various RP systems, it is possible to benefit from this technique in different dental practices, particularly in implementing dental prostheses for different applications. With research and development on a variety of RP systems and correspondingly built materials, it is possible to generate different kinds of dental prostheses for different applications. 

The limited confines of the RP technology include the high cost of the tools, complicated machinery engaged and dependency on an expertise to run the machinery during production. The authors believe that RP techniques are increasingly playing an imperative role in prosthodontics and will become one of the mainstream technologies for digital fabrication of dental prostheses in near future. 
